# Zen and the brain: mutually illuminating topics

**DOI:** 10.3389/fpsyg.2013.00784

**Published:** 2013-10-24

**Authors:** James H. Austin

**Affiliations:** Department of Neurology, University of Colorado Denver School of MedicineDenver, CO, USA

**Keywords:** Zen, meditation, egocentric, allocentric, thalamic model of enlightenment

## Abstract

Zen Buddhist meditative practices emphasize the long-term, mindful training of attention and awareness during one's ordinary daily-life activities, the shedding of egocentric behaviors, and the skillful application of one's innate compassionate resources of insight-wisdom toward others and oneself. This review focuses on how such a comprehensive approach to training the brain could relate to a distinctive flavor of Zen: its emphasis on *direct experience*, with special reference to those major acute states of awakening that create deep transformations of consciousness and behavior. In Japanese, these advanced states are called *kensho* and *satori*. Ten key concepts are reviewed. They begin by distinguishing between the concentrative and receptive forms of meditation, noticing the complementary ways that they each train our normal “top–down” and “bottom–up” modes of attentive processing. Additional concepts distinguish between our two major processing pathways. The self-centered, egocentric frame of reference processes information in relation to our body (our *soma*) or to our mental functions (our *psyche*). The other-centered frame of reference processes information anonymously. Its prefix, *allo*- simply means “other” in Greek. Subsequent concepts consider how these useful Greek words—ego/allo, soma/psyche—correlate with the normal functional anatomy of important thalamo ↔ cortical connections. A plausible model then envisions how a triggering stimulus that captures attention could prompt the reticular nucleus to release GABA; how its *selective* inhibition of the dorsal thalamus could then block both our higher somatic and psychic cortical functions; so as to: (a) delete the maladaptive aspects of selfhood, while also (b) releasing the direct, all-inclusive, globally-unified experience of other. Two final concepts consider how the long-term meditative training of intuitive functions relates to certain kinds of word-free spatial tasks that involve insightful creative problem-solving.

Only those contents of consciousness can be developed that correspond to the organization of the brain.

Walter R. Hess (1881–1973)

## Introduction

These words by Hess suggest that the following pages will invoke neurophysiological explanations, not loose metaphysical notions. That said, this review proposes that neuroscientists and Zen trainees can learn a few things from each other's models of consciousness. What does Zen training emphasize during its long-term approach to meditation? First, the mindful training of attention and awareness during one's *ordinary* daily life activities. Second, shedding the layers of maladaptive habits, overly self-centered attitudes and behaviors that waste time and energy. Third, enhancing one's innate, intuitive resources of insight-wisdom. Fourth, applying these fresh insights skillfully, with increasing compassion, both toward all other beings and one's own well-being.

These emphases are part of a long path of brain training that can lead toward more adaptive traits of character. The path emerges along that broad interface between two crucial domains, self and other. The former represents the distinctive interior consciousness of our personal self. The latter refers to that other consciousness representing the environment outside our skin. An important aspect of this path is epitomized in the historical account of a man, originally called Siddhartha. The records indicate that his behavior was substantially transformed after he emerged from a major state of awakened consciousness. Thereafter, he would be known as the “enlightened” one, the Buddha.

During the four decades since Hess died, the neurosciences have continued to learn more about the organization of the brain. Still, some readers may question: is it appropriate in this special issue of Frontiers for a neurologist to speculate about how such an acute episode could have transformed a 35-year old man like Siddhartha? On the other hand, only secular explanations will be tentatively proposed. They will address fundamental issues at the crucial interface between self and other. In order to focus on such an interface, this review leaves to other contributors the complex topic of correlating meditation with neuroimaging data. Left to its closing pages is the discussion of subtle incremental deconditionings of the maladaptive self, and a consideration of how such changes could emerge along a continuum of practical intuitive, creative, problem-solving *traits*. A glossary at the end helps to define useful terms.

The next pages summarize aspects of 10 key conceptual issues that are raised either in four books previously published or the one now in press. The Zen approach to training specifically targets unwarranted self-centeredness. It studiously avoids first-person references. This principle is not possible to achieve in this kind of review which necessarily refers to the author's several publications.

## Ten key conceptual issues

The first concepts summarize Zen meditative practice in terms of psychological phenomena. Later concepts suggest principles of neural organization that govern important physiological mechanisms. These mechanisms are involved both during our normal modes of ordinary consciousness, and in our normal modes of ordinary *sub*conscious processing. Moreover, many of their phenomena surface in the foreground of the kinds of advanced extraordinary states of consciousness that are often called “awakening” or “enlightenment.” The Japanese terms for these brief states are *kensho* and *satori*. Samples of frontier research will later be cited which appear to have fertile interdisciplinary implications.

### Zen meditative training

This meditative approach trains attention by focusing body and mind on the ongoing, *mindful* perception of each present moment as it really exists right now. Gradually, an increasingly calm awareness becomes the setting for the emergence of more subtle introspective memory skills. These are *automatic* “recollections”. They serve useful, self-correcting ends. (Austin, 2011, pp. 95–96, 98). Their subconscious meta-cognitive memory functions were always inherent in the original meaning of the ancient Pali term, *sati*. A newly-coined word, “remindfulness,” can serve a useful purpose. It simply acknowledges this involuntary, helpful overview memory capacity. (Austin, 2011, pp. 94–98) When Emerson pointed to the surge of this natural, insightful, affirmative mode of “guidance,” he used the apt phrase, “lowly listening” to describe its *re*mindful qualities (Austin, 2011, p. 145).

### The broad scope and depth of long-term Zen training

One doesn't “learn” Zen meditation in just a few days or weeks. The major target during the early months and years is one's self-centered *I-Me-Mine* complex and its unfruitful, maladaptive, emotional attachments. (Austin, 1998, pp. 43–47, 50–51; Austin, 2006, pp. 13–14) Long-term monastic training goes on to address the diverse existential, instinctual, and emotional aspects of the personality that cause unnecessary suffering. None of these ingrained egocentric attitudes surrender without a struggle. During meditative retreats (Austin, 1998, pp. 138–140), trainees benefit from many opportunities both to endure their own liabilities and to uncover their innate assets, while being guided by an authentic teacher (Austin, 1998, pp. 119–125; Austin, 2006, pp. 64–69).

### Three developments in the neurosciences have converged in recent decades

**Figure 1:**
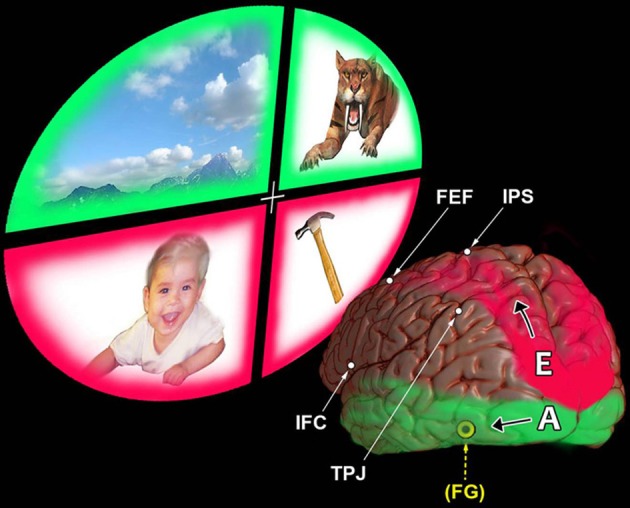
**Egocentric and allocentric attentive processing; major differences in their efficiencies.** This view contrasts our top–down dorsal *egocentric* networks with those other networks representing our ventral *allocentric*, bottom–up pathways. The reader's vantage point is from a position behind the *left* hemisphere, looking at the lower end of the occipital lobe. This person's brain is shown gazing up and off to the left into quadrants of scenery. The items here are imaginary. The baby and the hammer are within reach, in the space down close to the person's body. The scenery above and the tiger are off at a distance, out of reach. Starting at the top of the brain are the two modules of the *dorsal*, top–down attention system: the intraparietal sulcus (IPS) and the frontal eye field (FEF). They serve as the attentive vanguards linked with our subsequent sensory processing and goal-oriented executive behavior. Notice how they are overlapped by the upward trajectory of the *upper* parietal → frontal egocentric (E) system. This is a self-referential system. Its arching red pathway is shown as beginning in the upper occipital region. Notice that a similar red color also surrounds the *lower* visual quadrants containing the baby (at left) and the hammer (at right). Why? To indicate that this dorsal, “northern” attention system attends more efficiently—on a shorter path with a lesser “wiring cost”—to these *lower* visual quadrants. This enables our parietal lobe senses of *touch* and *proprioception* to “handle” easily such vitally important tangible items down close to our own body. In contrast, our two other modules for cortical attention reside lower down over the outside of the brain. They are the temporo-parietal junction (TPJ) and the regions of the inferior frontal cortex (IFC). During bottom–up attention, we activate these two modules of the ventral attention system chiefly on the *right* side of the brain. There, they can engage relatively easily the networks of allocentric processing nearby (A). The green color used to represent these *lower* temporal → frontal networks is also seen to surround the *upper* visual quadrants. Why? This is to suggest the ways this lower (“southern”) pathway is poised *globally* to use its two different specialized systems of pattern recognition. One is based on our sense of *vision*, the other on our sense of *audition*. Both serve not only to identify items off *at a distance* from our body but also to infuse them instantly with meaningful interpretations. The yellow FG in parenthesis points to this lower pathway's inclusion of the left fusiform gyrus. This region, hidden on the undersurface of the temporal lobe, contributes to complex visual associations, including our sense of colors.

- Neuroscience research has identified two basic network systems of attention operating at the cortical level. (Austin, 2009; Corbetta and Shulman, 2011; Kubit and Jack, 2013, pp. 29–34). Here, in a provisional sense, several aspects of their functional anatomy may be summarized when using such terms as *dorsal* and *ventral*, upper and lower, “top–down” and “bottom–up,” more voluntary and more involuntary.
  Research in meditators indicates that our dorsal attention system for top–down attention becomes more involved during the narrowly focused, *concentrative* meditative practices. (Austin, 2009, pp. 39–43) In contrast, the ventral attention system *orients itself* reflexively toward the subtler forms of bottom–up attention and global awareness. These become cultivated more gradually during the other kinds of meditative practices that are more openly *receptive*.
- Neuroscience research has also made major contributions to the neural correlates of self/other issues. Two major processing pathways have been identified, also dorsal and ventral in their early course. These networks provide consciousness with two anatomically separate versions of “reality” that are soon blended seamlessly (Austin, 2009, pp. 53–64). Our self-consciousness is most familiar with the “feeling” of the first version. Figure 1 illustrates how the dorsal pathway can tap into the specialized touch and proprioceptive resources inherent in the parietal lobe. This overlapping is convenient, because these senses of touch and proprioception help us manipulate important tangible items located inside that lower field of space lying *nearest* our body.
  The figure helps to appreciate that this upper, occipito-*parietal, egocentric* processing pathway follows a trajectory that is closest to, and overlaps, those same *lateral* and *superior* cortical regions which also represent the body schema of our *somatic*, physical self. So, can this upper pathway be described as asking only a *one*-word question: “Where?” No. A hungry person, seeing an apple within reach, instantly refers all lines of sight from this apple back to his or her own physical axis of self in order to grasp it. Therefore, this upper pathway seems poised to ask a much longer question. This highly practical question is structured in terms that correspond with the three dimensions of our personal space: “Where is that apple *in relation to Me and My body?*” (Austin, 2009, pp. 54–55, 58–59).
  The ventral pathway relies on other specialized refinements, those associated with its capacities for seeing and hearing. Notice the lower origin of this other cortical pathway. It begins in the inferior occipito-*temporal* region and extends toward the inferior frontal region. Such a trajectory confers a very different, *allocentric* perspective. (Austin, 2009, pp. 55–74) *Allo*, from the Greek, simply means “other.” Why is the existence of this hidden other-referential pathway so unfamiliar to us? Because such a silent “frame of reference” begins to operate *anonymously*. It asks the question, “*What* is that object?” Instantly, subconscious pattern-recognition skills identify that object. Simultaneously, other skills decode what that object *means*. (Austin, 2009, pp. 130–149) When Freud coined the useful diagnostic term, *agnosia*, he could then apply it to the isolated *loss* of visual meaning caused by a discrete lesion which had damaged a patient's temporal lobe (Austin, 2009, pp. 130–135).
  Our ordinary conscious processing remains unaware that it is deploying a seamless blend of these two complementary—upper and lower—categories of spatial reference. (Austin, 2009, pp. 57–58) Such ignorance vanishes *when the entire upper version drops out*. Zen parlance applies the following metaphor of selfless “emptiness” to this abrupt release from all former attachments to the intrusive self: “The bottom falls out and releases its bucket-full of water.” (Austin, 2009, pp. 191–193) *Now the lower version's allocentric processing stands unveiled*. Its innate subconscious capacities, perceived in isolation, are revealed for the first time. This “awakening”—this fresh perspective of “real” reality, infused with instant recognition and meaning—is an awesome surprize. (Austin, 2006, p. 361–371).
- Neuroscience research has also begun to attach a shorthand term, “default network,” to a large consortium of heterogeneous regions. The three largest regions occupy the *medial* prefrontal cortex, the *medial* posterior parietal cortex, and also the lateral cortex of the angular gyrus. Included within this triad and its several extensions are representations of both intrinsic, autobiographical and topographical association functions. (Austin, in press) The more rostral parts of this coalition contribute to an impression of personal sovereignty: our self as *the sole psychic* agency, its operations consistent with those of a private, autonomous *I-Me-Mine*. (Austin, 2009, pp. 74–75) Yet if each such mental notion about our personal self is to lodge in memory as a coherent event, it helps to encapsulate it within the context of some particular local scene. (Austin, 1998, pp. 390–391) These topographical, scenic details are essential if this locale is to serve as the “frame” enabling each event memory later to be used for purposes of navigation. (Austin, 2009, pp. 72–74) Only when each intimate episode is anchored both in one particular place in *our* own environment, and in one particular moment of *our* own “*time frame*” can these separate episodes become organized into a detailed, useful, lifetime, personal narrative.

How can any three-pound brain normally maintain such a life story, packed with notions that its omni-self is truly an ongoing, functioning agency? Not surprisingly, the early PET studies showed that the coalition of self-referential regions required an especially active ongoing metabolism even during seemingly “resting” conditions. Functional MRI signals arising from this consortium became even more activated (above such arbitrary “baselines”) when researchers assigned discrete self-referential tasks to their normal subjects. (Austin, 2006, pp. 204–207; Austin, 2009, pp. 75–76, 266–267) Subjects who tried to meditate in thought-free silence in the scanner discovered how much their “monkey-mind” wandered during the so-called “resting” state of quasi-“baseline” consciousness.

Importantly, fMRI signals from these same (mostly medial) intrinsic networks were acutely reduced (below baseline) at the instant that a sudden external stimulus event captured a subject's attention. Further fMRI research revealed a second important finding: the activity of these chiefly medial, self-referential cortical regions varied *inversely* with the activity of the lateral attention regions. (Austin, 2009, pp. 98–108) These separate self and attention regions co-participated—but in *opposite directions*—in the peaks and valleys of a slow, spontaneous, *endogenous* rhythm. This *reciprocal* rhythm required no external stimulus. It recurred *slowly*—around three times a minute—on its *own* largely independent cycle (Austin, 2011, pp. 32–34).

The Swiss physiologist, Walter Hess, was co-awarded the Nobel Prize in 1949. His pioneering research documented the dynamic switching capacities that lurked in the deep central diencephalic regions of the brain (Austin, 1998, pp. 190, 194, 232, 635–636). Extensions of that research make plausible today's hypothesis that some regions in the axial core of the brain—in and around the thalamus—might qualify as the deep origins for these reactive and spontaneous shifts that occur up in the recent cortical fMRI data. After all, these are fast and slow, switchings *on* and switchings *off*. They are enlisting separate cortical regions that make crucial commitments *either* to attention *or* to the representations of the self. Moreover, these regions respond not only within both hemispheres simultaneously, but also in a *reciprocal* manner (Austin, 2006, pp. 197–198, 427–428). Were Hess alive today, he would surely be encouraging neuroscientists to identify which basic mechanisms *organize* these two remarkable anticorrelated physiological feats.

### Major awakened states are experienced as selfless

The annals of Zen demonstrate more than an early, historical emphasis on the mindful training of attention and awareness (Austin, 1998, pp. 69–73). They also document numerous examples during which a sudden external stimulus—like the unexpected “CAW” of a crow—served not only to instantly capture attention but also to precipitate states of awakening (Austin, 1998, pp. 452–457; Austin, 2006, pp. 303–306; Austin, 2009, pp. 109–117; Austin, 2014, Chapters 5, 6). So, one wonders: could Siddhartha's legendary “awakening,” two and a half millennia ago, also represent a comparable event? Indeed, it was said that this state of enlightenment was triggered, before dawn, when he looked *up* and saw the brilliant “morning star” (Austin, 2011, pp. 77, 209). The ancient astronomers were familiar with this bright celestial object. They recognized the obvious fact that it traveled on a path, unlike a star. They gave the name, Venus, to this distinctive planet whose brilliance still lights up our Eastern sky before sunrise.

Explicit statements about selflessness entered the Buddha's earliest discourses. Examples of no-self sentences are found in two of the ancient Udana sutras: “When no you remains, then this, *just this*, will be the end of your suffering.” “Getting free of the conceit that “I am” is truly the ultimate happiness.” (Austin, 2014, Chapter 1; Austin, 2011, vi) Scientists have reason to be skeptical: could any rigorous common ground ever link old Buddhist legends with neurobiology? (Austin, 1998, pp. 1–7, 677–683). On the other hand, might what neuroscience is learning about the neurophysiological origins of selfhood help explain how *selflessness* could occur?

### Our normal unified thalamo-cortical connectivities

**Figure 2:**
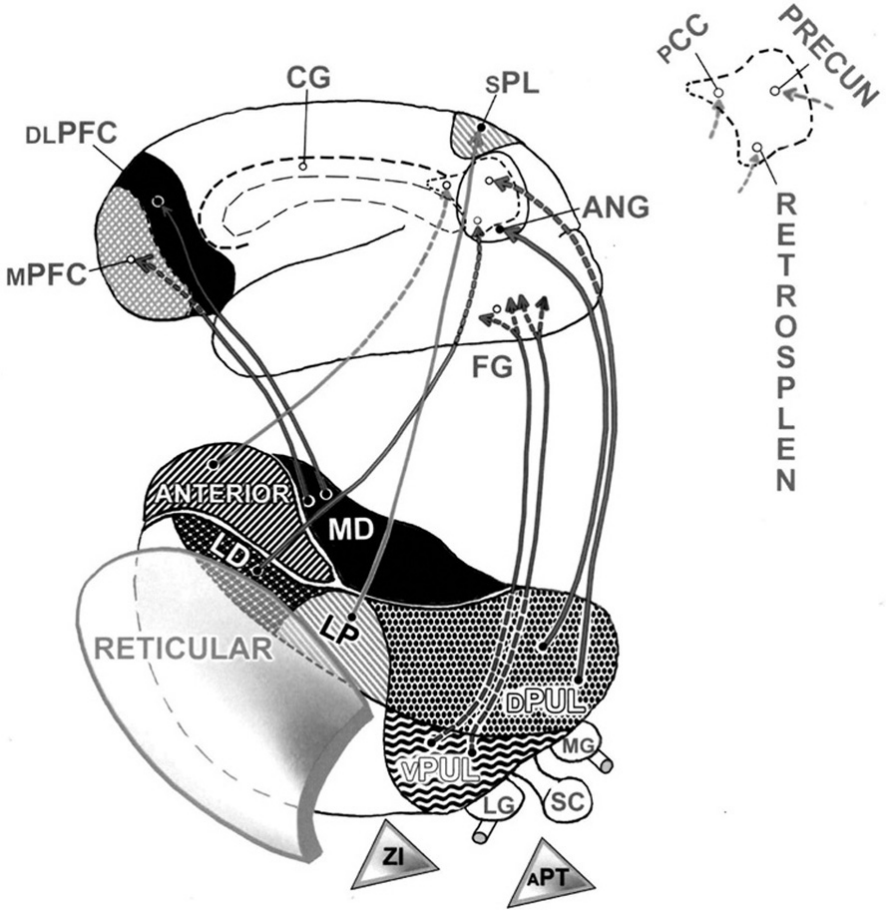
**Thalamocortical contributions to the dorsal egocentric and ventral allocentric processing streams.** This composite view shows the pathways (normally bi-directional) which connect the thalamus with the cortex. For convenience in viewing, only left-sided structures are shown. These connections supply key regions both on the outside and inside of the left hemisphere. Pathways predominate from all five nuclei in the dorsal tier of thalamic nuclei. Up front are the three limbic nuclei. The *medial dorsal* (MD) thalamic nucleus projects to the prefrontal cortex, both to its medial (MPFC) and to its outer, dorsolateral (DLPFC) surfaces. The deep medial area of cortex in the back of the brain is shown enlarged at the top right. Here, the projections from the two other adjacent dorsal nuclei of the limbic thalamus can also be seen reaching this medial cortical surface (dashed lines). Thus, the *anterior thalamic nucleus* projects to the *posterior cingulate cortex* (PCC) (a major connection hub), and the *lateral dorsal nucleus* (LD) projects to the *retrosplenial cortex* (RETROSPLEN), a major memory-based resource for recollection. Down at the bottom right, one path leads up from the *dorsal* pulvinar (DPUL) to the *angular* gyrus (ANG) on the *outer* cortical surface. Dashed lines indicate that a second path from this dorsal pulvinar leads up to the *precuneus* (PRECUN) on the *medial* parietal surface. Other projections from the *lateral posterior* (LP) nucleus supply the *superior parietal lobule* (SPL), our major somatosensory *association* region. These connections help to anchor each person's subliminal sensory impression: I exist as an independent, tangible, physically-articulated body schema. Relatively few pathways are shown emerging from the thalamus to serve the lower, allocentric processing stream. However, messages from the *ventral pulvinar* (VPUL) would pass first through the region of the *fusiform gyrus* (FG) on the undersurface of the temporal lobe. These associations ramify, to become further refined on their way forward and upward through the other-referential networks in the rest of the temporal lobe that lead on toward the frontal lobe. The figure shows three important GABA inhibitory nuclei, artificially detached at the bottom. They are the *reticular nucleus*, the *zona incerta* (ZI), and the *anterior pretectal nucleus* (APT). Two sensory relay nuclei of the thalamus are also shown at the bottom right. The *lateral geniculate nucleus* (LG) relays visual data to the occipital cortex. The *medial geniculate nucleus* (MG) relays auditory information to the auditory cortex. The *superior colliculus* (SC) in the midbrain relays its reflexive visual and related polymodal messages quickly through both the dorsal and ventral pulvinar to the cortex. Its counterpart, the inferior colliculus, plays a similar auditory role. Not shown are two *somatic* sensory relay nuclei in the ventral tier. These medial and lateral divisions of the ventral posterior nucleus lie in front of the ventral pulvinar. They relay sensation from the head and body, respectively. *cingulate gyrus* (CG).

Intricate interactive circuitries merge thalamic functions with those of our cortex (Austin, 2006, pp. 167–176). What is special about the three *limbic* nuclei in the front of our dorsal thalamus? Why could they play a crucial role in modulating these to-and-fro, rapidly oscillating connections between thalamus and cortex? Because these are the three thalamic nuclei poised to relay *over-emotionalized* messages from our limbic system up to pertinent parts of our self-referential cortex and to receive reverberations from them. Indeed, these three limbic nuclei interact with most of those same medial cortical association regions that item 3C had just singled out. Recall that these key regions of neocortex appear to be organized in a manner that can represent major attributes consistent with the autobiographical aspects of our psychic self that are linked to its topographical memories (Austin, 2009, pp. 87–94) (Please see Figure 2).

Figure 2 illustrates which limbic nuclei and cortical regions are organized to become co-activated either during an acute surging emotional overload or during subsequent un-checked ongoing ruminations (Austin, 1998, pp. 347–352, 567–570; Austin, 2006, pp. 239–265, 396–398; Austin, 2009, pp. 223–247; Austin, 2011, pp. 160–162). They include: (a) the *medial dorsal* nucleus and its several connections with the *medial prefrontal cortex* in particular; (b) the *anterior* thalamic nucleus and its connections, including those with the *posterior cingulate* cortex in particular; (c) the *lateral dorsal* nucleus and its connections with the event-memory-linked topographical and navigational functions of the *retrosplenial* cortex.

The thalamus serves as an important “pacemaker” for the brain. All of our sensations ascend through its nuclei (except for smell) on their way up to our cortex. Yet these bi-directional thalamo ↔ cortical connections, over-simplified here in Figures 1, 2, only hint at some explanations for how we might *normally* represent our omni-self's ordinary perceptions, emotions, and its journal entries into long-term memories. The question remains: how could a brain drop off the *deep* layers of the maladaptive self during the awakened states of kensho and satori?

### The inhibitory role of the reticular nucleus of the thalamus and its extra-reticular allies

A thin layer of nerve cells caps the rounded contours of the thalamus (Austin, 1998, pp. 267–271, 591, 605; Austin, 2006, pp. 176–178). This is the *reticular nucleus*. Existing fMRI data don't list it. Do not be misled. Its neurons release GABA (gamma aminobutyric acid), a *powerful inhibitory* neurotransmitter. Could this GABA play a “self-annihilating” role? Yes. How? By virtue of its selective capacities to readjust the synchrony of our usual thalamo ↔ cortical oscillations (Minn, 2010; Austin, 2011, pp. 35–37). Included in this selectivity are the capacities to dissociate visual functions in the ventral and dorsal streams (Austin, 2014, Chapter 11).

A simple analogy helps illustrate how the release of GABA might shift our waking consciousness away from its usual, dominant self-centered role. Try on a set of active, noise-canceling headphones. They generate a profile of sound-wave oscillations. These are 180° out of phase with those frequencies causing undesirable background noise. When the headphones are switched on, their selective tuning accomplishes two goals simultaneously: you hear the music coming through clearly; you block out the loud rumble of distracting traffic noise.

The reticular nucleus does not act alone. It has two, lower level, extra-reticular inhibitory allies (Austin, 2006, pp. 178–179) (Figure 2). The *zona incerta* releases its GABA on higher-order thalamic nuclei (including the medial dorsal nucleus). The *anterior pretectal* nucleus projects to both the medial dorsal and lateral dorsal nuclei of the limbic thalamus. It also stands poised to modulate the diversity of relevant functions among the somatosensory, intralaminar, and other nuclei that are included within the ventral and medial thalamus (Sherman and Guillery, 2013).

### An instructive Zen “mini-koan”: which direction do you face, when you want to move straight “ahead”?

Most self-orientations operate automatically. A covert motivation urges our first leaning forward. Instantly—too fast for thought—it commits us to “head” in one particular direction (Austin, 2006, pp. 106–108). If such subconscious systems are to resolve the riddle posed above, they first need to “know” where our own head is already located, and be capable of using visual information from our eyes that confirms the direction toward which the rest of our face is assumed to be pointing.

In short, we're using a subconscious, direction-sensitive, personal “behavioral compass” (Austin, 2006, pp. 172–174). Why might two nuclei of the limbic thalamus (anterior [dorsal] and lateral dorsal) be assigned some role in establishing this covert behavioral compass? Perhaps because this subcortical network does more than add to our static notions of being a private, interior self. Perhaps it could also be preparing us to lean, move, and navigate. How? By accessing subconscious links to hidden memories, to reconstructions of past events that had “taken place,” as we say, in the scenery *outside* our skin. We had encoded useful personal/local details during these remote prior events (Austin, 1998, pp. 390–391; Austin, 2009, pp. 259–260). These had served to frame the topographical signature representing each new, memorable spatial environment.

Recent fMRI evidence indicates how we retrieve two such events from memory (Elman et al., 2013). One event is newly-learned; the other is old and familiar. The newly-learned event was the outside appearance of one particular building. When subjects retrieved this building from recent memory they activated their *anterior* angular gyrus, supra-marginal gyrus, posterior cingulate and posterior precuneus (Austin, 2009, pp. 252). However, they activated different regions when they retrieved a long-familiar location from memory. This was a different building, the appearance of which was linked to intimately personalized resonances. In contrast, this more meaningful, self-centered, older recollection correlated with different activations. These occurred in the *posterior* angular gyrus (along the *ego*centric, E, parietal processing pathway of Figure 1), and in the lateral occipital cortex. Moreover, the medial regions now activated included the *anterior* precuneus *and* retrosplenial cortex, as well as the parahippocampal gyrus and the medial prefrontal cortex. Emerging from such subliminal topographical contrasts is the suggestion that when *I* become familiar with a place, *I* tend to regard it almost as though it had become *part of Me and was on My* “turf.”

### Different roles for the dorsal and the ventral thalamic nuclei during extraordinary states of consciousness

Item 5 explained how the five nuclei of the dorsal thalamus contribute to the normal overly-dominant egocentric attitudes of our psyche and soma. Item 6 then drew attention to selective capacities of deep inhibitory systems. It suggested that these GABA systems could have the potential to dissolve one's psychic and somatic association functions from the field of consciousness during *kensho*. The words, *anatta*, no-self, and non-*I* are among the standard terms used to describe the resulting selfless attributes of this advanced state (Austin, 2009, pp. 118–121). This abrupt dissolution at the core of the sense of self is noteworthy for two reasons. It can help explain why the deep roots of primal fear also drop out of kensho's field of waking consciousness, as does all personal sense of the passage of time (*achronia*) (Austin, 2006, pp. 378–383; Austin, 2009, pp. 193–196).

What other networks could remain active in a brain during kensho? What evidence offers a potential explanation for the fresh impression of unity that prevails throughout the whole “new” field of conscious experience? Leading the list of candidates would be the brain's lower allocentric pathways. Their “other” frame of reference is not a new category of experience. This anonymous perspective had always been there, its contributions playing only a hidden, subordinate role. (A in Figure 1) Now liberated from the dominant subjectivities of the former intrusive self, these other-referential resources could be openly expressed in an undiluted, disinhibited manner. Such a release from prior suppression could then be further enhanced in the course of a disinhibitory rebound (Austin, 2006, pp. 416, 418, 421).

Different observers bring an array of cultural and personal expectations to the vast topic of “enlightenment” (Boyle, 2013). Suppose a curious reader were to press one neurologist-witness to distill the 18 characteristics of kensho's awakening into only two simplified sentences (Austin, 1998, pp. 542–544). The language—strange on any page—might sound something like this:

- In empty anonymity, this now-unveiled other-referential mode is liberated into the foreground of consciousness, reifying the perfection of the whole mental field with a meaningful global objectivity beyond reach of mere words (Austin, 2006, pp. 329–333, 383–387).
- An astonishingly fresh impression of immanent reality prevails throughout this non-dual state of “Oneness”: all things, seen selflessly in the total absence of fear, are comprehended “as *they* really are.”

What seems to vanish in kensho's fresh perspective? Certainly not the world in 3D. What vanishes is only the former self's intrusive sense of sovereignty over it. When the former egocentric self no longer remains the “owner” of its ongoing perceptions, then the environment opens up to reveal percepts that are experienced with utmost “objectivity” (Austin, 1998, pp. 43–47, 263–267, 591–621; Austin, 2006, pp. 327–356, 414–432; Austin, 2009, pp. 85–94; Austin, 2011, pp. 163–165).

Shodo Harada Roshi (2000) aptly describes such a major transformation. It is an emptiness that cannot be conceptualized: “a state of being empty of ego, but full of what can come through [i.e., allo-perception] when that ego has been let go of.”

Do not conclude that the gradual diminution of the ego during decades of authentic Zen training will remove that pragmatic sense of self which enables one to resolve life's complexities by adapting to them in an increasingly mature, realistic, matter-of-fact way. Rather is the training oriented toward dissolving those negative, neurotic distortions of ego defenses imposed by one's overconditioned self-centered attachments (Austin, 1998, pp. 34–36). Longitudinal multidisciplinary studies will be required in order to specify which precise sequences of neural mechanisms need to mature in order for genuine wisdom and compassion to evolve. This caveat holds both for the kinds of decades-long research required to study normal control populations (Vaillant, 2012) or a carefully-matched cohort of long-term meditators (Austin, 2006, pp. 352–356, 399–401; Austin, 2009, pp. 221–262).

A different model of organization and reorganization is proposed for that alternate state of consciousness termed internal absorption (Austin, 1998, pp. 469–479; Austin, 2010). Internal absorption represents a preliminary state, one not uncommon during the early years among trainees who mediate regularly. Paradoxical phenomena enter its intensified awareness: vision “sees” into a vast, pitch-black ambient space; audition “hears” the “sound of absolute silence,” perception loses every *physical* sense of self from head to toe. In a quest for preliminary explanations of internal absorption, its agenda of 16 descriptors again leads us to the frontiers of neuroscience research.

A plausible model for such sensate phenomena can also begin on the basis of a GABA blockade (Austin, 1998, pp. 589–590; Austin, 2006, pp. 313–322; Austin, 2009, pp. 98). This inhibition could be applied to the lowermost regions in the back of the thalamus. (Please refer to Figure 2) Inhibition down here could prevent impulses from rising up to the cortex through the lowermost ventral sensory *relay* nuclei. They include the lateral geniculate, medial geniculate, ventral posterior medial and lateral nuclei, respectively.

### A descriptive continuum of intuition: ordinary creative insights at one end; extraordinary states of awakened insight-wisdom off toward the other

Carl Jung (1875–1961) understood the key role of intuition. It supplies, he said, that “superior analysis, insight or knowledge which consciousness had not been able to produce” (Jung, 1969; Austin, 1998, pp. 545–553). Could long-term meditative training do more than cultivate a person's attention and affirmative traits of character? Could it also influence subconscious, intuitive mechanisms that subtly enhance a person's *flexible* creative problem-solving behavior? (Austin, 2003, 2014, Chapter 14).

Two recent experiments by Strick et al. (2012) are germane. The 63 meditators in their first experiment had completed 6 months to 5 years of prior Zen practice. In the evening, between 6 and 9pm, one group (led by a Zen master) then meditated for 20 min. The second Zen group served as controls. They relaxed at the same time, without meditating, also for 20 min. Both groups then went to individual cubicles. There they performed three sets of five Remote Associates Tests using a computer screen. (For example, given three words, search your associations for that one fourth word which they all share in common.) The subjects who had just meditated solved more test items (7.00) than did those who had merely relaxed (5.94), *p* = 0.02.

In the second experiment, the response times of 32 Zen meditators were measured during similar word association tests. Again, the subjects who had just meditated solved more Remote Associates Test items than did their controls (6.82 vs. 4.87), *p* < 0.01. They also solved them faster (taking only 13.22 vs. 16.37 s), *p* < 0.05. In addition, the groups were then asked to free associate to a new collection of different questions. However, in this instance, each of the 20 questions might have not just one, but three or four possible answers. (e.g., “Name one of the four seasons.”) Moreover, this time—*before* each question—a priming word-answer appeared *subliminally* on the screen (e.g., “Summer”). No subject could “see” this hidden, priming word consciously, because it lasted only 16 ms.

Now the question was: could meditation unveil any of their *subconscious* sensitivities? If so, would this hidden awareness enable the subliminal priming word to reshape the answer? The meditators' answers did match the hidden priming words at the *p* = 0.06 level, just short of statistical significance. In contrast, the relaxed control group showed no priming effect.

The Remote Associates Test is often interpreted as a task for verbal creativity that combines an initial divergent search with convergence functions. Therefore, the experiments suggest that these particular Zen meditators (tested in the *evening*) showed evidence consistent with an enhancement of creative processing after having meditated (Austin, 2009, pp. 125–130, 154–188). Why should researchers in the future specify the hours during which they conduct their cognitive experiments? Because many normal subjects may not reach their performance maximum for working consciousness until the later hours between 7 and 9 pm (Austin, 1998, pp. 338–347). In this regard, Shannon et al. (2013) made an intriguing observation. In normal subjects, the medial temporal regions show greater local fMRI connectivities in the morning hours. In the evening hours, more connections open up that will link the frontal and parietal neocortex with the striatum and brain stem.

In their study of creativity, Colzato et al. (2012) interpreted this Remote Associates Task as a way to index the competence of convergent thinking, and assessed the productivity of divergent thinking with the Alternate Uses Task. Their 19 meditators had been practicing mixtures of concentrative and receptive forms of meditation for an average of 2.2 years. The task for each subject was to spend only 35 min a day (on each three separate days) either in concentrative meditation, or in receptive meditation, or in a baseline control condition. The data suggested that 35 min of focused meditation (only) did not sustain convergent thinking processes toward a single solution, but that a separate 35 min of receptive meditation did support divergent thinking processes.

Our normal ordinary, intuitive quests for meaning become successful only when we repeatedly apply subtle convergent and divergent attentive processing mechanisms with appropriate flexibility. Many of these flexible interactions integrate the functions of fronto-temporal and fronto-parietal lobe networks in particular (Austin, 2009, pp. 130–173). However, diverse phenomena unfold quickly during advanced alternate states of consciousness. They present unusual blendings of different attributes. The two sentences condensed in item 8 suggest that the flash of *selfless* insight-wisdom (Skt: *prajna*) is extraordinary for two reasons. First, for the way it dissolves self-centered processing; second, for the way it liberates other functions that are consistent with enhanced modes of allocentric attentive processing (Austin, 2009, pp. 178–188, 199–214). The egocentric vacancy in the core of the psyche leaves an extraordinary impression: all root origins of selfhood and deep natural survival angsts seem to have dropped off. This acute, ineffable release from the deepest instincts of primal fear is especially liberating (Austin, 1998, pp. 569–570; Austin, 2006, pp. 232–237, 357–387; Austin, 2014, Chapter 14).

### Relevant evidence from recent experiments in non-meditating subjects

Insight *happens*. Insight is not driven by logic-tight sequences of deliberate thought. For centuries, Zen masters emphasized that advanced degrees of insight (Skt: prajna) played a key transforming role in the Buddhist meditative Path. The masters also warned their trainees: steer clear of those heavy burdens imposed by word language, ruminating thoughts, and fixed conceptual barriers (Austin, 2009, pp. 150–152; Austin, 2014, Chapters 3, 6, 8). Does any contemporary research into the pros and cons of language appear to support such an orthodox empirical stance?

The first task for the normal subjects studied by Bergen et al. (2007) was straightforward: listen to words that would be spoken in the form of simple, short, recorded sentences. The whole meaning of each sentence hinged on where, in space, each event could have taken place. The two possibilities were either higher *up*, or lower *down*, in the extrinsic environment. For example, in some sentences the subjects could hear an up-word (“sky”) being spoken. Other sentences would specify a down-word (“grass”). The subjects' next task was a simple visual discrimination: they indicated by a button press whether they saw a circle or a square. Each visual target appeared either at a higher, or lower place on the computer screen. They could see each ◦ or □ clearly, during a long 200 ms interval. Some subjects took 30 ms *longer* to signal this discrimination. What caused this slowing? Why had they hesitated? Notably, the delay occurred when they saw either this square, or this circle, in that very *same*—upper, or lower—location, the same place which had just been inferred by that up-or-down word-language inserted into the prior sentence. When did these prior spatial nouns or verbs cause the greater interference delays? When both the visual and the auditory processing converged during those tasks that were chiefly referable to the *upper* visual fields. Figure 1 suggests that these upper visual fields are represented by the lower occipital ↔ temporal lobe pathways.

How do words interfere with other brain functions? Could word entanglements that arise among language networks (perhaps especially in our left hemisphere) sometimes compete for neural resources with nearby intuitive processing mechanisms? Could such obstacles, acting either directly or indirectly, block the free-flowing access to adjacent networks that might otherwise help us express innate degrees of selfless, insight-wisdom?

In this regard, a different kind of experiment has examined the ways the right and left hemispheres function in normal subjects (Chi and Snyder, 2011, 2012). In these studies, the brain is being stimulated by the gentle flow of *direct* current. This technical approach is called *transcranial direct current stimulation* (tDCS). This direct current is delivered at low amperage to the *scalp* over the right and left anterior temporal regions, *simultaneously*. However, the left anterior temporal scalp electrode serves as the negative pole (–). This cathodal pole tends to *hyper*polarize the resting potentials of nerve cells in the underlying left anterior temporal lobe. This makes these left nerve cells *less* excitable. In contrast, the right anterior temporal scalp electrode serves as the + pole. This anodal pole tends to *de*polarize the resting potentials of the underlying nerve cells on the right side. What is the net result of these modulations? Notice that this result generates the asymmetrical expression of temporal lobe functions. Those pathways on the right side become *more* readily excitable. Those on the left side—the side dominant for language—become *less* excitable (Austin, 2014, Chapter 13).

These normal subjects received transcranial direct current stimulation to the scalp in this manner for only 10–17 min. Next they were challenged to solve either some difficult “matchstick” tests (Austin, 2009, pp. 183–184) or to solve another, very difficult, “connect-the-9-dots” test. In brief, the data showed that tDCS substantially improved the subjects' creative problem-solving performance for up to the next hour beyond that which occurred either in the sham controls, or when the directions of current flow were reversed. These results have implications both for the kinds of complications introduced by our words and for the nature of the kinds of insights we use during creative processing.

## In summary

It turns out that old Greek words, plus a few old words used in Zen, overlap with concepts recently evolving in neuroscience. The results can be mutually illuminating *if* their correlations are interpreted with appropriate caution. A longitudinal perspective is essential. Thirty-five year old Siddhartha had devoted six long years to a rigorous spiritual quest before he happened to glance *up* before dawn at that legendary morning star (Austin, 1998, pp. 7–8).

Is it feasible to attempt to develop a theoretical neural basis for such an “awakening” of insight-wisdom? The 10 topics just reviewed in this condensed version are the latest sample emerging from several decades spent exploring testable hypotheses (Austin, 1998, xvi; Austin, 2006, xvi; Austin, 2009, xvi; Austin, 2011, pp. 169–177). Some of these have the potential to clarify thorny issues increasingly important to meditators, researchers, and to society in general (Austin, 2012, in press).

### Conflict of interest statement

The author declares that the research was conducted in the absence of any commercial or financial relationships that could be construed as a potential conflict of interest.

## Glossary

Achronia: A state of consciousness lacking all sense of time, resulting in the impression of eternity.
Allocentric: Other-referential. The term serves as a useful contrast to our dominant mode of egocentric (*self*-centered) consciousness. Additional terms emphasize the anonymity implicit in allocentric processing. They include: stimulus-centered, object-centered, observer-independent.
I-Me-Mine: A descriptive psychological construct. It helps define the personal self in operational terms.
Kensho: Seeing into the essence of reality. The beginning of true long-term meditative training; a prelude to the depths of satori.
Koan: An enigmatic statement serving as a device for concentration.
Limbic thalamus: The three most rostral nuclei in the dorsal thalamus: the medial dorsal, anterior, and lateral dorsal nuclei. They are intimately connected both with the limbic system and with particular regions of the overlying cortex.
Prajna (Skt): The flashing insight-wisdom of enlightenment.
Remindfulness: The overview intuitive functions that tap into memory to provide us with subconscious forms of affirmative guidance. These normal recollection functions are implicit in an ancient Pali word, *sati*, that is usually translated only as mindfulness.
Reticular nucleus of the thalamus: The thin outer layer of GABA nerve cells capping the dorsal thalamus in particular. It plays a pivotal, normal inhibitory role in shaping our thalamo ↔ cortical interactions.
State: A temporary condition involving mentation, emotion, or behavior.
Trait: A distinctive ongoing quality of attitude, character or behavior. Traits can be transformed incrementally in the course of long-term meditative training, especially when reinforced by deep insightful *states* of awakening.
Zen: A form of Mahayana Buddhism which emphasizes a systematic approach to meditative training and to the development of character. Its two major schools—Rinzai and Soto—developed in China when Indian Buddhism evolved in the cultures of Taoism and Confucianism, and later spread to Japan.
